# Supplementary material for: Factors associated with differential seropositivity to Leptospira interrogans and Leptospira kirschneri in a high transmission urban setting for leptospirosis in Brazil
Source: medRxiv. 2023 Apr 11:2023.04.10.23288388. Preprint. [Version 1] doi: 10.1101/2023.04.10.23288388 (PMC10120806; doi:10.1101/2023.04.10.23288388)
Supplement: 1 [file NIHPP2023.04.10.23288388v1-supplement-1.pdf]

medRxiv preprint doi: https://doi.org/10.1101/2023.04.10.23288388; this version posted April 12, 2023. The copyright holder for this preprint (which was not certified by peer review) is the author/funder, who has granted medRxiv a license to display the preprint in perpetuity.
It is made available under a CC-BY 4.0 International license .

Table S1. Bivariate models for seroprevalence of *Leptospira kirschneri* Cynopteri (3522C) and *Leptospira interrogans* Icterohaemorrhagiae (Fiocruz L1-130).

	Cynopteri (3522C)			Icterohaemorrhagiae (Fiocruz L1-130)		
**Characteristic**	**N**	**OR[1]**	**95% CI[1]**	**N**	**OR[1]**	**95% CI[1]**
**Individual**						
Median Age (years)	2,600	1.03	1.02, 1.05	2,763	1.03	1.02, 1.04
Sex	2,600			2,763		
Male		—	—		—	—
Female		1.07	0.59, 1.99		0.69	0.52, 0.91
Ethnicity	2,562			2,724		
Brown		—	—		—	—
Black		0.86	0.47, 1.58		0.97	0.72, 1.30
White		0.36	0.02, 1.75		1.21	0.64, 2.11
Others		0			0	
Education	2,600			2,763		
5 or less		—	—		—	—
5 to 9		0.6	0.26, 1.34		1.09	0.76, 1.57
10 to 12		0.75	0.35, 1.61		0.95	0.66, 1.38
Higher Education		0.86	0.05, 4.40		0.43	0.07, 1.43
Never studied		3.64	1.35, 8.98		1.43	0.69, 2.73
Employment	2,600			2,763		
No		—	—		—	—
Yes		0.87	0.45, 1.62		1.73	1.30, 2.29
Risk occupation	2,600			2,763		
No		—	—		—	—
Yes		0.96	0.23, 2.67		2.72	1.81, 3.99
**Risk exposures**						
Walk barefoot	2,599			2,762		
No		—	—		—	—
Yes		0.75	0.39, 1.39		0.88	0.65, 1.18
Use of boots	2,597			2,760		
No		—	—		—	—
Yes		0.75	0.30, 1.58		1.22	0.86, 1.69
Cleaned sewage	2,286			2,448		
No		—	—		—	—
Yes		1.3	0.44, 3.05		2.55	1.75, 3.65
Sewage contact	2,599			2,762		
No		—	—		—	—
Yes		1.38	0.68, 2.62		1.4	1.01, 1.92
Open sewer	2,598			2,761		
No		—	—		—	—
Yes		1.32	0.71, 2.40		1.26	0.94, 1.69

medRxiv preprint doi: https://doi.org/10.1101/2023.04.10.23288388; this version posted April 12, 2023. The copyright holder for this preprint (which was not certified by peer review) is the author/funder, who has granted medRxiv a license to display the preprint in perpetuity.
It is made available under a CC-BY 4.0 International license .

Access to the house is paved?	2,600			2,763		
No		—	—		—	—
Yes		1.03	0.54, 2.14		1.03	0.74, 1.44
Wall material?	2,600			2,763		
No		—	—		—	—
Yes		1.1	0.38, 2.57		1.6	1.06, 2.36
**Presence of animals in the household**						
Presence of Cats	2,600			2,763		
No		—	—		—	—
Yes		1.89	1.01, 3.44		1.09	0.78, 1.50
Presence of Dogs	2,600			2,763		
No		—	—		—	—
Yes		0.86	0.45, 1.57		0.94	0.70, 1.25
Presence of Chickens	2,600			2,763		
No		—	—		—	—
Yes		1.46	0.35, 4.09		1.71	0.96, 2.85

[1]OR = Odds Ratio, CI = Confidence Interval
